# Extreme Viral Partitioning in a Marine-Derived High Arctic Lake

**DOI:** 10.1128/mSphere.00334-20

**Published:** 2020-05-13

**Authors:** Myriam Labbé, Catherine Girard, Warwick F. Vincent, Alexander I. Culley

**Affiliations:** a Département de biochimie, de microbiologie et de bio-informatique, Université Laval, Quebec City, Quebec, Canada; b Center for Northern Studies (CEN), Université Laval, Quebec City, Quebec, Canada; c Takuvik Joint International Laboratory, Université Laval, Quebec City, Quebec, Canada; d Institut de biologie intégrative et des systèmes (IBIS), Université Laval, Quebec City, Quebec, Canada; e Département de biologie, Université Laval, Quebec City, Quebec, Canada; National Institute of Advanced Industrial Science and Technology

**Keywords:** aquatic viral ecology, limnology, polar science, viromics

## Abstract

High-latitude, perennially stratified (meromictic) lakes are likely to be especially vulnerable to climate warming because of the importance of ice in maintaining their water column structure and associated distribution of microbial communities. This study aimed to characterize viral abundance, diversity, and distribution in a meromictic lake of marine origin on the far northern coast of Ellesmere Island, in the Canadian High Arctic. We collected triplicate samples for double-stranded DNA (dsDNA) viromics from five depths that encompassed the major features of the lake, as determined by limnological profiling of the water column. Viral abundance and virus-to-prokaryote ratios were highest at greater depths, while bacterial and cyanobacterial counts were greatest in the surface waters. The viral communities from each zone of the lake defined by salinity, temperature, and dissolved oxygen concentrations were markedly distinct, suggesting that there was little exchange of viral types among lake strata. Ten viral assembled genomes were obtained from our libraries, and these also segregated with depth. This well-defined structure of viral communities was consistent with that of potential hosts. Viruses from the monimolimnion, a deep layer of ancient Arctic Ocean seawater, were more diverse and relatively abundant, with few similarities to available viral sequences. The Lake A viral communities also differed from published records from the Arctic Ocean and meromictic Ace Lake in Antarctica. This first characterization of viral diversity from this sentinel environment underscores the microbial richness and complexity of an ecosystem type that is increasingly exposed to major perturbations in the fast-changing Arctic.

**IMPORTANCE** The Arctic is warming at an accelerating pace, and the rise in temperature has increasing impacts on the Arctic biome. Lakes are integrators of their surroundings and thus excellent sentinels of environmental change. Despite their importance in the regulation of key microbial processes, viruses remain largely uncharacterized in Arctic lacustrine environments. We sampled a highly stratified meromictic lake near the northern limit of the Canadian High Arctic, a region in rapid transition due to climate change. We found that the different layers of the lake harbored viral communities that were strikingly dissimilar and highly divergent from known viruses. Viruses were more abundant in the deepest part of the lake containing ancient Arctic Ocean seawater that was trapped during glacial retreat and were genomically unlike any viruses previously described. This research demonstrates the complexity and novelty of viral communities in an environment that is vulnerable to ongoing perturbation.

## Abstract (precis)

The Arctic is warming at an accelerating pace, and the rise in temperature has increasing impacts on the Arctic biome. Lakes are integrators of their surroundings and thus excellent sentinels of environmental change. Despite their importance in the regulation of key microbial processes, viruses remain largely uncharacterized in Arctic lacustrine environments. We sampled a highly stratified meromictic lake near the northern limit of the Canadian High Arctic, a region in rapid transition due to climate change. We found that the different layers of the lake harbored viral communities that were strikingly dissimilar and highly divergent from known viruses. Viruses were more abundant in the deepest part of the lake containing ancient Arctic Ocean seawater that was trapped during glacial retreat and were genomically unlike any viruses previously described. This research demonstrates the complexity and novelty of viral communities in an environment that is vulnerable to ongoing perturbation.

## INTRODUCTION

The Arctic is experiencing a disproportionate and accelerated rate of warming relative to the rest of the world (1), and this is resulting in profound changes throughout the North Polar Region (2). Among the ecosystems particularly sensitive to climate change are polar lakes because of the integral role that their ice cover plays in their structure and functioning (3). This is especially evident in the Canadian High Arctic, where lakes that were perennially covered in ice in the past are now ice free and in that state for increasing periods (4). One type of lacustrine environment that is considered a model ecosystem to study the impact of these changes is represented by meromictic lakes (3). These ecosystems are found at both poles and are characterized by stable, permanently stratified water columns. This persistent layering results in sharp biogeochemical gradients, which in turn create a wide range of microbial niches that are particularly sensitive to perturbation (5).

**FIG 1:**
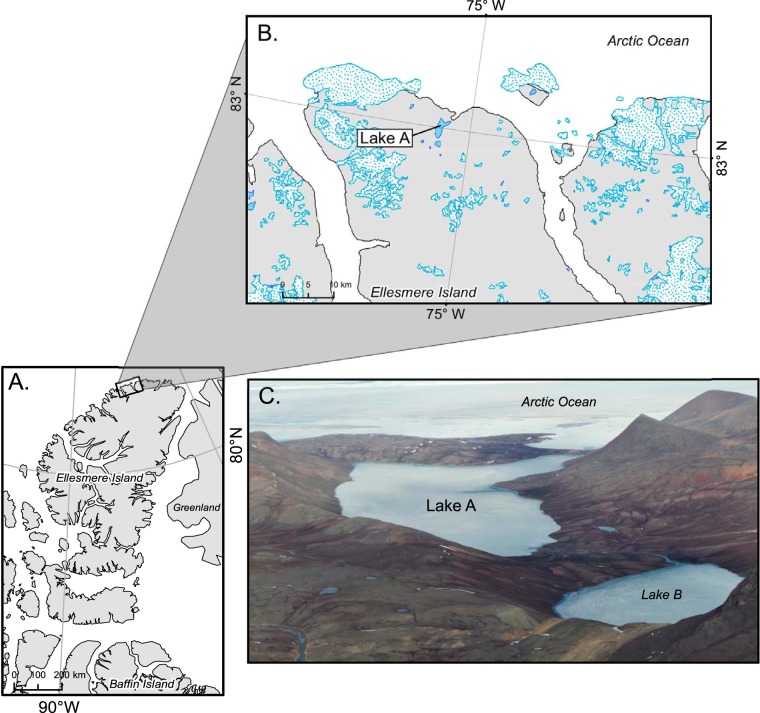
Map of the sampling site showing (A) Ellesmere Island in the Canadian High Arctic (Nunavut), (B) the location of Lake A on the northern coast of the island, and (C) a photograph of Lake A and part of its water catchment (photograph from Alexander Culley; reproduced with permission). Map credit: Michel Paquette, Département de géographie, U. de Montréal.

Lake A, on the far northern coast of Ellesmere Island in the Canadian High Arctic, is a meromictic lake that has received much limnological attention since its discovery in the 1960s (Fig. 1). This lake formed approximately 3,000 years ago when Arctic seawater was trapped by isostatic rebound and then overlaid with freshwater derived from melting snow and ice (6). The lake appears to have been perennially covered with ice throughout much of its history, with a temporary period of ice-out (the disappearance of ice from the surface of a body of water [such as a lake] as a result of thawing) in the 1940s (7). Over the past 2 decades, ice-free incidents have occurred with increasing frequency (3). The 128-m water column of Lake A is highly stratified and is comprised of a high-light, low-conductivity, oxygenated surface layer derived from snowmelt (the mixolimnion), a transition zone (the metalimnion), and a low-light, high-conductivity, anoxic stratum of bottom water that originates from the ancient Arctic Ocean (the monimolimnion) (8).

The pronounced gradients in light, temperature, conductivity, and oxygen in Lake A result in favorable habitats for diverse communities of microbes. In general, the prokaryote and microbial eukaryote communities in the mixolimnion differ entirely from those of the monimolimnion. The most important primary producers in the mixolimnion are chrysophytes and chlorophytes (9) and picocyanobacteria in the genus *Synechococcus* (10, 11). On the basis of amplicon sequencing, Comeau et al. (12) reported that *Proteobacteria*, *Actinobacteria*, and *Cyanobacteria* represent the abundant prokaryotes in surface waters. Pouliot et al. (13) found that the dominant archaeal representatives are affiliated with the *Euryarchaeota* except for the *Nitrosopumilus* (*Thaumarcheota*), a potentially important contributor to nitrification. In contrast, the largest contributors to primary production in the monimolimnion are green sulfur bacteria (GSB) (10). Other abundant bacteria in the monimolimnion include deltaproteobacteria and bacteria related to the marine SAR406 clade (12), and the dominant archaea are members of the phylum *Euryarchaeota* (13). Although the diversity of microbial communities of Lake A is strongly shaped by environmental conditions, those communities are also subject to predation by grazers and parasitism by fungi (14) and viruses. With the exception of the characterization of a cyanophage that was isolated from a pooled sample of High Arctic lakes that included Lake A (15), nothing is known about the Lake A viral assemblages and their impact on the microbial communities that they infect.

Viruses are pervasive, prolific, and active components of aquatic environments (16–18). They regulate the abundance and diversity of the organisms that they infect (19), influence the functional potential and evolution of their hosts by facilitating the transfer of genetic material (20), and ultimately may influence the availability of carbon and other critical nutrients (21). Yet viruses in the polar regions have received little attention. Previous studies mostly focused on the Arctic Ocean and Antarctica, and few have used metagenomics to investigate viral diversity (22), with a recent notable exception (23). A study of six lakes and ponds in the island of Spitsbergen in the Svalbard region of Norway used metagenomics to reveal that most of the viral sequences examined had no similarity to those in public databases (24). Among the sequences that were identified, most were related to DNA viruses with small, single-stranded genomes (24). However, these libraries were produced with an amplification method that preferentially amplifies single-stranded DNA (ssDNA), producing a biased portrait of viral diversity (25). This finding is consistent with results from Lake Torneträsk, a subarctic lake in northern Sweden (26). A comparison of the Svalbard lake viromes with viromes from several subarctic and temperate environments showed that Arctic sequences tended to cluster together, suggesting a certain degree of endemism in lacustrine Arctic virus communities (26).

Viruses have been detected in a variety of lakes in the south polar region, including freshwater Limnopolar Lake in the Antarctic Peninsula region (27). The most relevant data for the present study are from Ace Lake, a meromictic lake in the Vestfold Hills of East Antarctica. Like its northern counterpart Lake A, Ace Lake is a well-characterized polar meromictic lake that has been studied over the past 40 years. Ace Lake, like Lake A, is typically covered by thick ice and has a permanently stratified water column with steep physical and chemical gradients (5). The community structure of Ace Lake also resembles that of Lake A, with autotrophic protists, aerobic heterotrophic bacteria, and abundant picocyanobacteria in its mixolimnion and anaerobic prokaryotes in its monimolimnion, including photosynthetic GSB, sulfate-reducing bacteria, fermentative bacteria, and methanogenic archaea (previously reviewed by Laybourn-Parry and Bell [5]). Viral abundance in the surface waters of Ace Lake remained relatively stable throughout all seasons, ranging from 1 × 10^7^ to 6 × 10^7^ virions per ml (28). Abundances declined markedly in the oxycline and rose to reach peak concentrations in the monimolimnion (29). A comprehensive study of Ace Lake microbial interactions based on metagenomics and metaproteomics found a partitioning of viral taxa between the mixolimnion and monimolimnion, with high relative abundances of protist-infecting phycodnaviruses and their virophages (30) above the oxycline and a high relative abundance of temperate bacteriophages belonging to the order *Caudovirales* below (29). Nevertheless, the points of similarity of Ace Lake’s viral ecology to that of Lake A remain unknown. As a sentinel of accelerating climate change in the Arctic, the Lake A microbial ecosystem needs to be fully defined, and establishing its viral diversity and distribution is important.

Our aim in the present study was to characterize the extracellular double-stranded DNA viral community of Lake A. We hypothesized that the distinct physicochemical properties and host communities in the three strata of the lake would result in pronounced differences in viral diversity among these layers. We also aimed to identify the viral assemblages associated with particular host groups and environmental conditions and to evaluate the similarity of Lake A viruses to viral communities elsewhere. We sampled the under-ice water column in midsummer and used a minimally invasive filter-based approach to collect viral particles from replicate samples at multiple depths. These samples were then extracted and sequenced to produce viromic data for analysis.

## RESULTS

### Physical, biological, and chemical properties of the water column.

**FIG 2:**
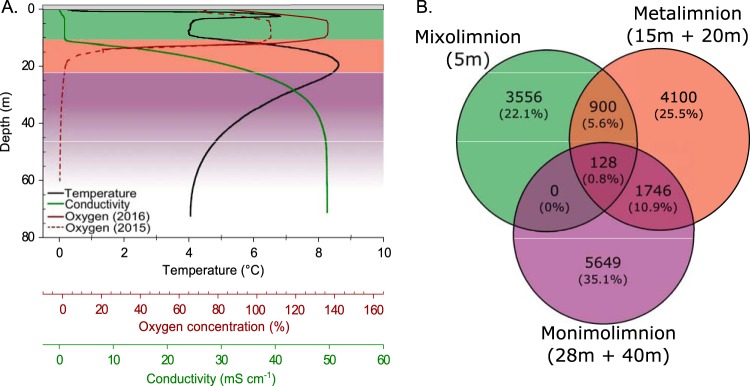
Chemical and biological stratification of the Lake A water column. (A) Profiles of temperature (black), specific conductivity (green), and oxygen concentrations (red). The oxygen profile represents a combination of measurements below 22 m from 2015 (dotted line) and 2016 (unbroken line). Bottom waters (monimolimnion) were anoxic, with an oxygen concentration of 0% at 22 m. The light gray rectangle represents the 92 cm of lake ice cover. (B) Venn diagram showing the proportions of vOTUs shared among the lake strata. The percentage of shared vOTUs for each section is shown in parentheses. In both figures, the three strata of the lake are indicated by color as follows: green, mixolimnion; red, metalimnion; purple, monimolimnion.

The surface waters (mixolimnion) of Lake A were fresh and highly oxygenated, with low specific conductivities (Fig. 2A; see also Table 1). There was a sharp transition zone (metalimnion) between 11 and 22 m, with an increase in conductivity and a pronounced maximum in temperature of 8.5°C at 19 m (Fig. 2A; see also Table 1). Oxygen concentrations dropped rapidly with depth across the metalimnion, and data from the previous year showed that the concentration drops to zero at approximately 22 m. The bottom stratum (monimolimnion) exhibited seawater conductivity values (>40 mS cm^−1^) (Fig. 2A; see also Table 1).

**TABLE 1:** Physicochemical characteristics of the three strata in Lake A

Stratum	Depth interval (m)	Temp (°C)	Dissolved O_2_ (%)	Conductivity (mS cm^−1^)
Mixolimnion	0–11	0.4–6.8	84–137	0.1–1.4
Metalimnion	11–22	5.3–8.5	124–0.1^*a*^	1.4–36
Monimolimnion	22–bottom	4.0–8.5	0.00^*a*^	36–50

a Values based on 2015 data.

Dissolved organic carbon concentrations (DOC) ranged from 1.8 to 5.0 mg liter^−1^, with the highest values in the 20-m sample. Both total nitrogen (TN) and total phosphorus (TP) increased with depth. TN reached a maximum concentration of 8.67 mg liter^−1^ in the monimolimnion. TP was below detection limits for depths 15, 20, and 28 m but reached 743 μg liter^−1^ at 40 m (Table 2). Pearson correlation analysis was used to identify closely related environmental variables that could confound further analysis of the data (see Fig. S1 in the supplemental material). The results indicated that TP was closely correlated with TN, and TP was therefore removed from further analyses, as were dissolved inorganic carbon, pH, and fucoxanthin concentration. Chlorophyll-*a* (chl-*a*) was present throughout the water column, while bacteriochlorophyll-*e* (bchl-*e*) and bchl-*e*-like pigments, indicative of GSB, were detected only below 20 m. The highest concentration of the carotenoid zeaxanthin, a proxy for cyanobacteria, was at the surface, with lower concentrations at greater depths (Table 2).

**TABLE 2:** Limnological characteristics of sampled depths^*a*^

Depth (m)	Water chemistry			Pigment concn			Microbial abundance					VPR
	DOC (mg liter^1^)	TP (μg liter^−1^)	TN (μg liter^−1^)	Chl-*a* (μg liter^−1^)	Bchl-*e* (μg liter^−1^)	Zeaxanthin (μg liter^−1^)	Photosynthetic eukaryotes (10^3^ ml^−1^)	Green sulfur bacteria (10^3^ ml^−1^)	Picocyanobacteria (10^3^ ml^−1^)	Heterotrophic bacteria (10^3^ ml^−1^)	Viruses (10^4^ ml^−1^)	
5	1.8	17	113	0.25 (9)	<LOD	0.15 (1.3)	1.62 (39)	NA	43.7 (8)	813 (6)	435 (23)	5.1
15	3.2	<25	172	0.21 (6)	<LOD	0.13 (14)	0.88 (13.4)	NA	71.1 (29)	261 (13)	96 (24)	2.9
20	5.0	<35	303	0.18 (7)	1.92 (53)	0.08 (7)	1	88	47.6 (11)	189 (3)	113 (48)	4.8
28	4.2	<45	4,401	0.24 (34)	20.5 (27)	0.07 (40)	2	183	18.1 (19)	436 (16)	492 (11)	10.8
40	3.9	743	8,668	0.09 (12)	2.21 (9)	0.05 (11)	0.1	4	7.21 (9)	228 (4)	632 (5)	26.9

a Pigment concentration data are from HPLC analysis, and microbial abundance data are from flow cytometry. Each value represents the mean of results from experiments performed in triplicate (percent coefficient of variation [CV] data are indicated in parentheses). The analytical limits of detection (LOD) were as follows: 0.05 mg/liter for DOC; 5 μg/liter for TP; 20 μg/liter for TN; 0.004 μg/liter for Chl-*a*; 0.012 μg/liter for bacteriochlorophyll-*e* (Bchl*e*); and 0.047 μg/liter for zeaxanthin. NA, not applicable.

10.1128/mSphere.00334-20.1 FIG S1 Metadata autocorrelation matrix showing covariance between metadata collected from Lake A. The color-coded values are for Pearson correlation coefficients. Download FIG S1, PDF file, 0.2 MB. Copyright © 2020 Labbé et al. 2020 Labbé et al. This content is distributed under the terms of the Creative Commons Attribution 4.0 International license.

Heterotrophic bacteria were present throughout the water column and were most abundant at the surface, where they reached concentrations of approximately 10^6^ cells ml^−1^. Picocyanobacteria were, in general, an order of magnitude less abundant than other bacterial species, peaking at 15 m at 7.1 × 10^4^ cells ml^−1^. Autotrophic eukaryotes were present at 5 m and 15 m but were nearly absent in deeper samples. The opposite trend was apparent for the GSB. GSB were not detected in surface samples but were abundant at depth, with a peak level of 1.8 × 10^5^ at 28 m (Table 2). Viral particle concentrations ranged from 9 × 10^5^ to 6 × 10^6^ particles ml^−1^ and were lowest at 15 m and highest at 28 m.

### Viral diversity and community structure.

**FIG 3:**
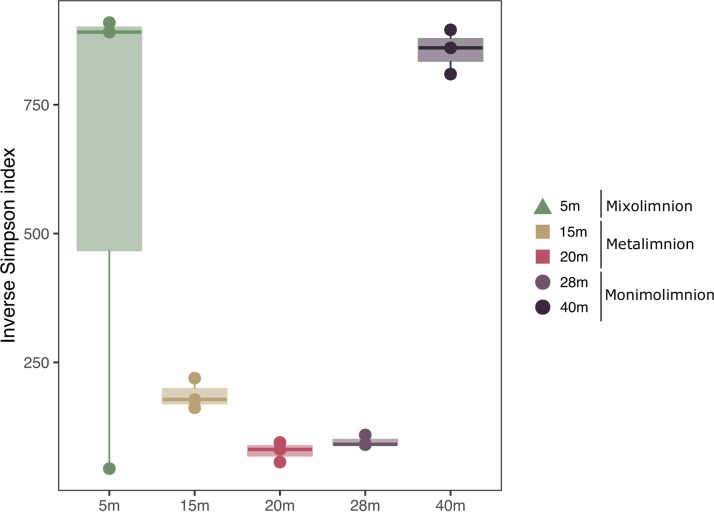
Within-sample viral diversity by depth assessed from the number of vOTUs in a particular cluster following read mapping. Note that this figure does not reflect the real diversity of viruses in Lake A due to sampling biases and differences in sequencing depths, among other factors. In addition, it is likely that the data indicating viral diversity at 40 m represent underestimates of the levels of diversity relative to the other depths, as the percentage of reads mapped to assembled contigs from this depth was markedly lower (Table S2). No significant differences were observed across depths (*P* = 0.122), likely due to the outlier at 5 m.

Processing of the viral metagenomic data yielded a total of 16,080 viral operational taxonomic units (vOTUs), which are considered here as individual sequences representing a group of highly similar viral contigs. Each stratum of the lake had a distinct set of vOTUs, with over 80% of the total vOTUs recruiting reads from only one layer: 22.1% from the mixolimnion, 25.5% from the metalimnion, and 35.1% from the monimolimnion (Fig. 2B). The divergence between lake strata was further indicated by the relatively low proportion of vOTUs shared between layers. While the metalimnion shared a limited number of vOTUs with both the mixolimnion and the monimolimnion, the few vOTUs shared between the mixolimnion and the monimolimnion were also detected in all strata of the lake (*n *= 128, 0.8% of vOTUs). The levels of diversity across depths and layers were not significantly different (Fig. 3, *P > *0.05).

**FIG 4:**
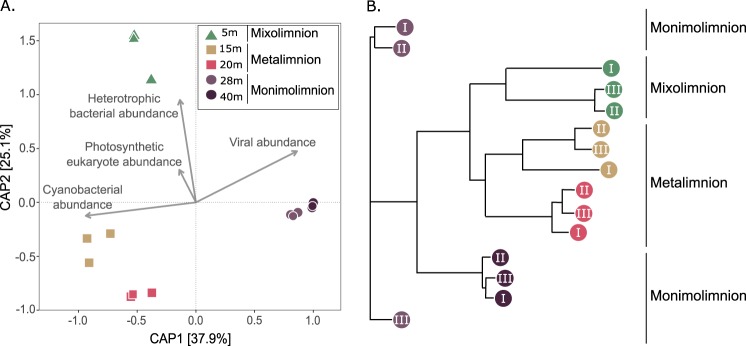
Viral diversity by depth. (A) Constrained analysis of principal coordinates (calculated with Bray-Curtis dissimilarity), including flow cytometry counts of major host groups and viruses. The proximity of a host group (vectors) to a particular viral community (shapes) is an indicator of the strength of the association. (B) Neighbor-joining tree of all depths and replicates color coded by depth.

The viral communities were examined by constrained analysis of principal coordinates (CAP) using Bray-Curtis dissimilarity. The first two axes explained 63% of the variation (CAP1 = 37.9%, CAP2 = 25.1%). Samples from a given depth were highly similar, with nonsignificant dispersal within triplicates (*P > *0.05, Fig. 4A). However, viral communities from different depths and different lake strata accounted for significant amounts of the variations in the data set (87% and 64%, respectively, *P < *0.001). Among the three strata, the mixolimnion and metalimnion samples grouped more closely together (Fig. 4B). An analysis of CAP space representing vOTU communities and groups of microbes on the basis of flow cytometry counts showed an association between photosynthetic eukaryotes and heterotrophic bacteria and vOTU communities in the mixolimnion, between cyanobacteria and vOTUs from 15 m, and between virus-like particles and vOTUs in the monimolimnion (Fig. 4A).

Weighted gene coexpression network analysis (WGCNA) grouped cooccurring vOTUs into 28 modules that contained from 43 to 3,832 vOTUs per module. Some of these modules were significantly correlated (*P < *0.001) with several environmental properties, including oxygen concentration (module C), TN concentration (module O), and bchl-*e* concentration (modules T and V; Fig. S2). Also, each lake stratum was correlated with specific modules (see Table S1 in the supplemental material): modules B and C with the mixolimnion, modules I and J with the metalimnion, and modules O and U with the monimolimnion. We then compared the vOTUs from these modules with viral sequences from the IMG/VR metagenomic database. The percentages of sequences from the modules that were identified ranged from 1% to 37% (see Fig. 6) on the basis of our search criteria (defined below).

10.1128/mSphere.00334-20.2 FIG S2 Module correlation to environmental parameters from the WGCNA package showing Pearson correlation scores from −1 to 1 between each module and each environmental parameter (Bonferroni-corrected *P* values are in parentheses). Rectangles in bold highlight significant correlations (*P* < 0.001) between a module and a lake stratum, while dotted rectangles highlight significant correlations between modules and chemical and biological variables. Download FIG S2, PDF file, 0.2 MB. Copyright © 2020 Labbé et al. 2020 Labbé et al. This content is distributed under the terms of the Creative Commons Attribution 4.0 International license.

10.1128/mSphere.00334-20.5 TABLE S1 Modules with significant correlations to environmental variables based on WGCNA. Download Table S1, PDF file, 0.1 MB. Copyright © 2020 Labbé et al. 2020 Labbé et al. This content is distributed under the terms of the Creative Commons Attribution 4.0 International license.

### Annotation of viral assembled genomes.

**FIG 5:**
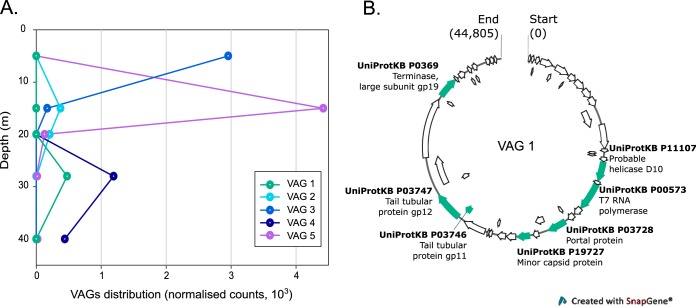
(A) Distribution of 5 ecologically relevant viral assembled genomes (VAGs) in the water column (normalized counts as obtained from Read2RefMapper). (B) Genomic map of VAG 1. Open reading frames (ORFs) corresponding to UniRef90 protein clusters are labeled and shown in turquoise.

We identified 504 circular contigs in the database. The circularity of the sequences suggests that they were complete viral genomes, and we refer to these below as VAGs (viral assembled genomes). Among these, we focused on VAGs that were identified as viral in origin with confidence (VirSorter category 1 or 2), were larger than 30 kb, contained hallmark viral genes, and were part of a WGCNA module that was significantly correlated with an important limnological variable such as the concentration of bchl-*e* (a proxy for GSB) or a particular stratum of the lake, for example. Our analysis of the 10 VAGs that best fit these criteria is summarized in Table S3. Among the 10 VAGs, few to no known genes (less than eight per genome) were identified with Prokka (based on Swiss-Prot, a manually curated database), although those genes that were identified were viral genes from viruses in the order *Caudovirales*. In the case of VAG 1 (Fig. 5B), six genes were found to correspond to *Enterobacteria* phage T7 and one to *Enterobacteria* phage T5, including hallmark structural proteins, a terminase, a helicase, and an RNA polymerase. We used the program ViPTree (31) to further classify the VAGs (Fig. S4). On the basis of this analysis, most of the viruses were found to be closely related to viruses in the family *Myoviridae* or the family *Siphoviridae* and to infect hosts in the phylum *Firmicutes*. Overall, however, the VAGs were only distantly related to the nearest taxon. We selected five VAGs based on the depth of annotation, correlation with environmental characteristics (WGCNA analysis, Fig. S2), and abundance in our samples and determined their distribution in the water-column (Fig. 5A; see genomic maps in Fig. S3). The VAGs were generally specific to a lake stratum or depth. For example, VAG 1 was found only at 28 m and VAG 5 was largely restricted to the metalimnion.

10.1128/mSphere.00334-20.3 FIG S3 Genome maps of four selected VAGs. (A) VAG 2: 106 open reading frames (ORFs), 1 nonhypothetical protein. (B) VAG 3: 33 ORFs, 1 nonhypothetical protein. (C) VAG 4: 76 ORFs, 2 nonhypothetical proteins. (D) VAG 5: 63 ORFs, only hypothetical proteins. Download FIG S3, PDF file, 0.1 MB. Copyright © 2020 Labbé et al. 2020 Labbé et al. This content is distributed under the terms of the Creative Commons Attribution 4.0 International license.

10.1128/mSphere.00334-20.4 FIG S4 Viral proteomic tree showing the 10 Lake A VAGs (described in more detail in Table S3) and 1,267 reference dsDNA viruses infecting prokaryotic hosts, using log-scaled branch length. Lake A VAGs are identified by red stars and red branches. Download FIG S4, PDF file, 0.2 MB. Copyright © 2020 Labbé et al. 2020 Labbé et al. This content is distributed under the terms of the Creative Commons Attribution 4.0 International license.

### Comparative viromic analysis.

**FIG 6:**
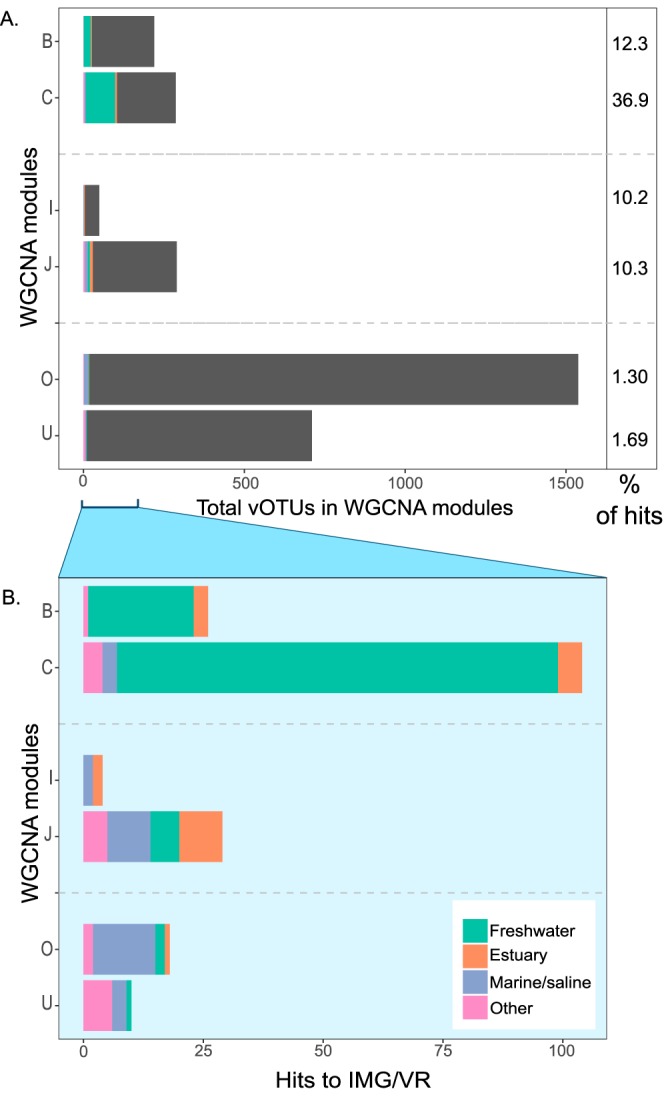
Similarity between WGCNA modules and habitat types. (A) Bar charts showing the habitats of origins of the sequences from the IMG/VR database that matched vOTUs from the WGCNA modules that were highly correlated (*P* < 0.001) to lake strata. The percentage of vOTUs with significant hits to the IMG/VR database is shown on the right for each module. The length of the horizontal column indicates the total number of vOTUs in the module. (B) The expanded part of the graph shows the associated habitat types of only those vOTUs with significant matches.

We compared the most highly correlated modules identified with WGCNA with sequences in the IMG/VR database (32). This analysis revealed that the majority of vOTUs showed little similarity to previously published sequences (Fig. 6). More similarities were found between vOTUs from modules B and C (associated with the mixolimnion) and sequences from freshwater habitats such as lakes, bogs, and rivers, while vOTUs from modules O and U (monimolimnion) were found to be more highly associated with viromes from marine/saline habitats and sediments. The number of hits seen with vOTUs from modules I and J (metalimnion) was equal to the number seen with viromes from marine/saline habitats and estuaries, although module J also had hits to viromes from freshwater and wastewater.

**FIG 7:**
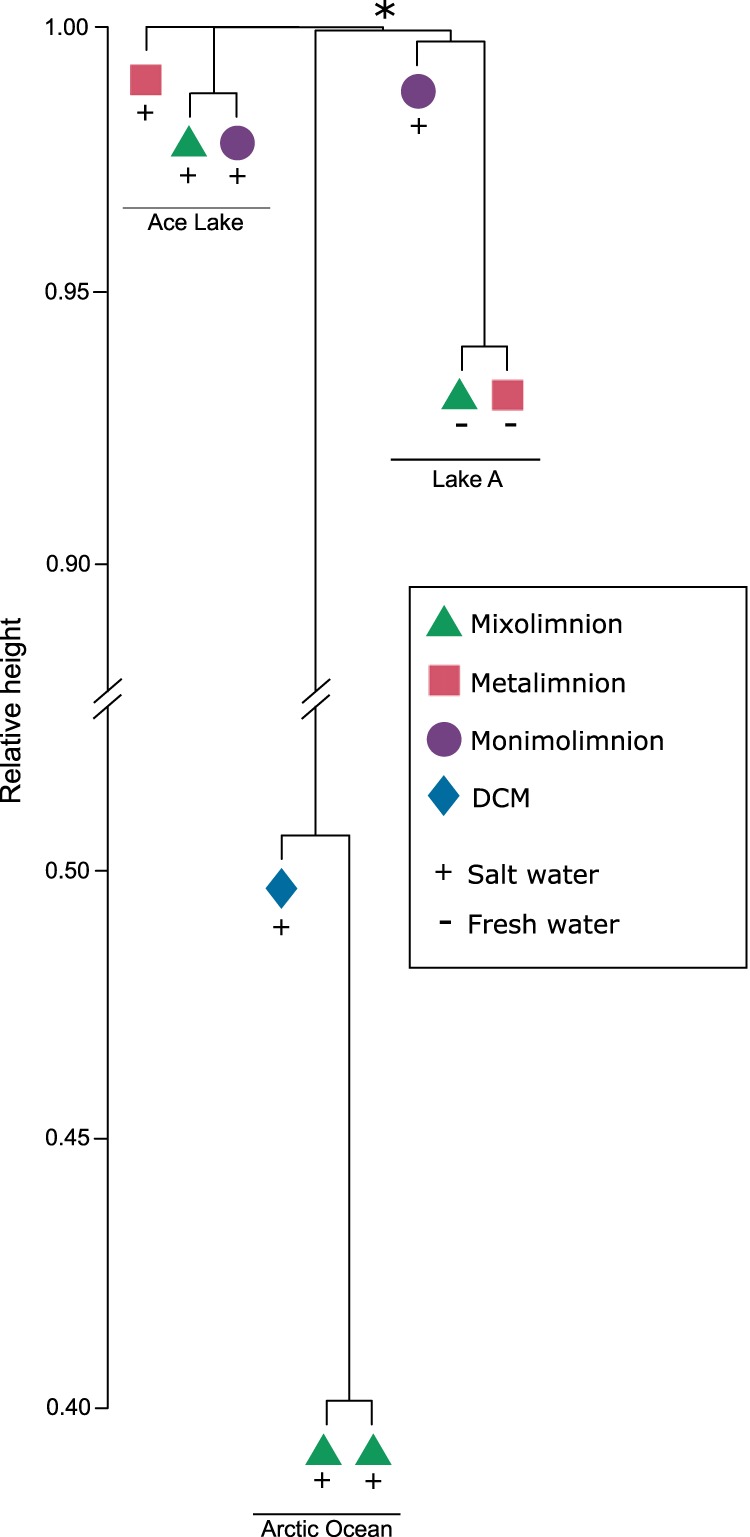
Comparison of Lake A, Ace Lake, and Arctic Ocean viral communities. The dendrogram shows Lake A data for depths 5 m (mixolimnion), 15 m (metalimnion), and 40 m (monimolimnion); Ace Lake data for depths 5 m (mixolimnion), 12.7 m (metalimnion), and 23 m (monimolimnion) (28); and three ocean samples from the Tara Polar expedition (station 196, 5 m; station 194, 5 m and 35 m) (33). The asterisk denotes the node of a clade that includes the Arctic Ocean and Lake A samples.

Finally, we compared viral reads from Lake A with viral sequences from two stations in the Arctic Ocean (33) and from Ace Lake, a meromictic lake in Antarctica (28), using Libra (34) (Fig. 7). On the basis of this analysis, the three Lake A strata formed an independent cluster within which the mixolimnion and metalimnion were most similar. Three samples from the Tara Polar project from two stations in the Arctic Ocean (station 196, 5 m; station 194, 5 m and 35 m) were also included (33), and these samples formed a distinct cluster (Fig. 7). The surface samples from the two stations formed a cluster despite the stations being separated by roughly 440 km, with samples from the DCM (deep chlorophyll maximum) zone being less closely related. The Lake A and Arctic Ocean samples formed a cluster with a basal node (identified with an asterisk in Fig. 7). The Ace Lake mixolimnion and monimolimnion grouped closely, while the metalimnion was more divergent.

## DISCUSSION

The vertical profiles of physical, biological, and chemical properties that we observed in Lake A (Fig. 2A) were comparable to those observed in studies of the site in previous years (7, 9, 11, 12, 14, 35), demonstrating that the lake continues to retain a vertical structure that is stable through time. The high concentrations of chl-*a* and zeaxanthin at the surface suggest a high abundance of picocyanobacteria of the genus *Synechococcus* in the mixolimnion, a distribution also observed previously by Antoniades et al. (10). Our cytometry data also confirmed an abundance of GSB at a depth of 28 m as also described previously by Van Hove et al. (11) and Comeau et al. (12), although a coincident peak of chl-*a* and the high counts of photosynthetic eukaryotes suggested that green sulfur bacteria might not be the only autotrophs at this depth. This pattern of abundant picocyanobacteria in the mixolimnion and abundant GSB in the monimolimnion was also observed in Ace Lake, Antarctica (5).

The marked partitioning of viral communities among the three lake strata observed in this study generally fits with previous studies of bacterial (10, 12), archaeal (13), protistan (9), and zooplanktonic (35) communities from Lake A. This segregation was most evident in the viral communities of the mixolimnion and monimolimnion (Fig. 2B) (see also Fig. 3 and 4B), where we found that virtually no virus types were shared (Fig. 2B). Viral community composition is determined by viral production and viral decay, which are determined in turn by abiotic factors (UV-B radiation, concentration of charged particles [36]) and biotic factors (host diversity, host metabolic state, predation, and extracellular enzymatic activity [37]). Consistent with our hypothesis, the distinct abiotic and biotic factors of the three lake strata have resulted in viral communities with striking differences in composition and diversity.

There are few studies that have compared levels of lake viral diversity over depth. A study from Lake Biwa (38) reported that >65% of viral genomes were unique to a particular depth. Similarly to Lake A data, a study of Lake Shunet, a sub-Arctic meromictic lake in Siberia (39), showed that very few viral taxa were present throughout the water column. However, in this study, the highest relative viral diversity was observed in the surface stratum. Unlike Lake A, Lake Shunet is not perennially covered in ice and thus has more exchange with the atmosphere. Seeding by aerosols may account for the relatively high microbial diversity at the surface. As has been found in most freshwater environments, including in this study, a majority (60 to 99%) of viral sequences were found to have no homology with sequences in published databases (40, 41). Although the data from lakes are few, the high relative percentage of unknown viral sequences in the monimolimnion and the extreme partitioning of viral communities between strata (<1% of vOTUs in common) in Lake A appear to be exceptional. It should be taken into consideration, however, that we applied stringent annotation criteria that could result in an overestimation of the number of unclassified sequences.

Studies investigating freshwater viromes have resulted in the observation of sharp differences in viral diversity driven by season (27, 42, 43) or by proximity to the shore or large water inputs or human perturbation (40, 44). Several studies have identified salinity as the primary driver of viral diversity (38, 45), where higher diversity corresponded with higher conductivity (45, 46). Watkins et al. (40) proposed that freshwater viral communities are likely to be more diverse than marine viral communities because freshwater environments are generally more heterogeneous. We did not find this to be the case with Lake A, where the viral assemblage in its unusual saltwater monimolimnion was potentially more diverse than in the less saline strata.

Heterotrophic bacteria and cyanobacteria dominated microbial cell counts at 5 and 15 m (Table 2), suggesting that bacteriophages and cyanophages comprise an important fraction of viral communities at these depths (Fig. 4A). Populations of protists, on the other hand, showed no clear association. This was to be expected since viruses that infect eukaryotes are generally less abundant than prokaryote-infecting viruses, reflecting the much lower concentrations of eukaryotic cells. Moreover, our study was limited to viruses with double-stranded DNA (dsDNA) genomes (there are more RNA viruses that infect eukaryotes than infect bacteria [47]), and our method included a prefiltration step (using a 0.22-μm-pore-size filter) that likely removed viruses of the putative order *Megavirales*, a widespread and abundant group of viruses infecting eukaryotes, with virion diameters that often exceed 220 nm (48).

Our results suggest that sedimentation is not an important driver of viral community composition in Lake A. The higher virus-to-prokaryote ratio (VPR) in the deep samples indicates that virus-host interactions in the mixolimnion are different from those in the monimolimnion. Factors that contribute to a higher VPR in the monimolimnion could include a lower level of exposure to UV radiation and a lower concentration of extracellular enzymes due to a lower abundance of prokaryotes, resulting in lower rates of viral decay and a longer extracellular virus residence time than at the surface (19). It is also possible that there is a higher proportion of lytic viruses (versus temperate viruses) in the monimolimnion than in the mixolimnion (49). A higher relative VPR in deeper waters has also been observed in Antarctic lakes (50) and in the deep ocean (51). In Ace Lake, Lauro et al. (29) observed markedly lower total virus counts and the presence of CRISPR-associated proteins at the depth with the highest abundance of GSB and postulated that GSB may be relatively more resistant to viral infection. However, we did not find the same trend in Lake A, where total virus abundance at the depth of the GSB peak was higher than at all but one other depth.

On the basis of WGCNA, we were able to identify groups of vOTUs (modules) that were associated with particular biogeochemical variables. One module (module C; see Fig. S2 in the supplemental material) was significantly correlated with high oxygen concentrations and may therefore have been enriched with viruses that infect oxygenic phototrophs such as cyanobacteria, although there were no modules that were significantly correlated with zeaxanthin concentrations, a proxy for cyanobacterial abundance. Two modules were significantly associated with the signature pigment of GSB (modules T and V, Fig. S2), which suggests that these modules were enriched with viruses that infect GSB. The WGCNA also identified modules that were significantly correlated with individual lake strata (modules B, C, I, J, O, and U, Fig. S2). We then compared the vOTUs from these modules with viral sequences from the IMG/VR metagenomic database. Consistent with the results of previous analyses of most environmental viromes (52), the vast majority of sequences from the modules were unidentified compared to IMG-VR at an 85% identity threshold. Less-stringent search criteria would likely result in a higher percentage of matches with known sequences, but our results generally reinforce the view that current viral sequence databases represent a small fraction of wild virus diversity. These results also suggest that the vOTUs from the monimolimnion modules, in particular, are highly divergent from those of known viral taxa, reflecting the unusual nature of this habitat.

The scarcity of genes that were identified in our subset of VAGs illustrates the challenges that viral ecologists face with wild viruses from underexplored environments. It should be noted that our annotation was based on matches to a manually curated database. This approach is likely more accurate but may have resulted in a lower percentage of matches than if other databases had been included. Several VAGs with potential ecological relevance (determined by association with environmental parameters) are present in our data set. VAG 1, for example, is found only at 28 m and belongs to a WGCNA module that correlates with the concentration of bchl *e*-like pigments and thus may infect the GSB that were found at high concentrations at this depth (11, 12), although our analysis did not link it with a *Chlorobium* host (Fig. S3). The (albeit limited) annotation of the VAG suggests that it belongs to the group of T-odd phages (Fig. 5B), but further analysis is needed. Several of the VAGs were classified in the *Siphoviridae*, which raises the possibility that they are capable of lysogeny (Fig. S3). However, we were unable to identify any of the genes typically associated with a temperate lifestyle.

We were able to compare the viral community compositions of two meromictic lakes, as well as that of Lake A’s monimolimnion (ancient Arctic Ocean seawater), with viral communities from the modern Arctic Ocean (Fig. 7). On the basis of our analysis, there was no overlap between the Arctic and Antarctic lakes. Although Lake A and Ace Lake share several physical and ecological properties, the lack of overlap is unsurprising given the pronounced differences in the histories and locations of the two ecosystems. Little overlap of viral communities from Arctic and Antarctic samples has also been observed (23, 43) at the species level (22). However, there may be convergence at higher taxonomic levels (22). Another factor to consider is that represented by the differences in methodology between the two studies, including differences in the sequencing platforms (454 pyrosequencing in Ace Lake versus Illumina HiSeq next-generation sequencing in this study) and sequencing depths, which can magnify small-scale variations and exaggerate divergences. This result is consistent with previous comparisons of microbiomes from both poles (23, 52, 53) and also with comparisons of the microbiomes of deep anoxic zones from different lakes (54). This demonstrates that the unique environmental conditions of a particular landscape can be the primary driver of the composition of the microbial communities in these aquatic environments.

Although a basal node is shared (Fig. 7) by the Lake A and Arctic Ocean samples, our analysis does not establish a well-supported link between the Lake A monimolimnion viral community and that in the present-day Arctic Ocean. This suggests that the genomes of viruses in Lake A water that were isolated from the Arctic Ocean several millennia ago are now highly divergent from those of their modern Arctic Ocean counterparts.

The two freshwater samples from the mixolimnion and metalimnion of Lake A formed a tight cluster that was distantly related to samples from brackish and saltwater samples. This result underlines the importance of salinity as a driver of viral diversity in this particular system. The monimolimnion of Lake A appears to contain a high diversity of vOTUs, a majority of which have no equivalent in current databases. This deep-water habitat is also especially vulnerable to climate change. Modeling of warming and ice-cover loss indicated that Lake A will lose its upper water column structure as the lake is increasingly exposed to wind-induced mixing (7). Studies of samples from Lake Romulus, a meromictic lake approximately 350 km south of Lake A, indicated that deep mixing will entrain salt at the surface, where the effects of freeze concentration may lead to even higher conductivity (11). Lake A will persist, but its chemical and physical habitat properties will likely be subjected to major perturbation, with effects on microbial communities, including viral abundance, structure, and diversity. The divergent viral community of the deep stratum may be especially vulnerable to future change.

### Conclusions.

The three distinct strata of Lake A, including the mixolimnion, metalimnion, and monimolimnion, harbored distinct viral communities and viral assembled genomes that shared few vOTUs, suggesting that viral export was a process of low significance. The highest abundance and highest relative diversity of viruses were in the monimolimnion, a layer of ancient Arctic Ocean seawater at the bottom of the lake. These viruses were mostly novel, indicating the need to better characterize the diversity of wild viruses and their functions, as well as the uniqueness of this habitat. Given that the Arctic is warming at an increasing rate, augmenting the vulnerability of Arctic stratified lacustrine environments such as Lake A, it is crucial to characterize the extant biota, including viruses, in these systems before they are irrevocably perturbed.

## MATERIALS AND METHODS

### Sample collection and processing.

Three holes spaced 10 m apart at a midlake site (lat 82.596679N, long 75.266029W; Fig. 1) were bored through the approximately 92 cm-thick ice cover of Lake A on 16 July 2016. Temperature, conductivity, and oxygen profiling of Lake A was done using a YSI 600QS sonde and an RBR Concerto conductivity, temperature, and depth (CTD) logger. Lake strata were identified based on the physicochemical profiles of the water column (Fig. 2A; see also Table 1). Water samples were then collected at five depths from the three lake strata in each of the three holes with a 7-liter Limnos water sampler as follows: mixolimnion, 5 m; metalimnion, 15 and 20 m; monimolimnion 28 and 40 m. The samples were collected in triplicate, using one replicate per hole. Water was then transferred in the field into Cubicontainers that had been cleaned with 2% (vol/vol) Contrad liquid detergent and 10% (vol/vol) American Chemical Society (ACS)-grade HCl (Sigma-Aldrich) and rinsed with lake water before sampling. Samples were kept cool and in the dark during transportation by helicopter to a field laboratory, where they were processed within 3 h of collection. Subsamples (preserved using a final concentration of 0.5% [vol/vol] glutaraldehyde [grade 1]; Sigma-Aldrich) were taken before filtration for flow cytometry counts and for determination of pigment concentrations (performed using filtration with 25-mm-diameter Whatman GF/F glass-ﬁber ﬁlters [nominal pore size, 0.7 μm] and immediate freezing at –20°C) and of levels of TP, TN, and DOC (stored at 4°C in the dark) (54). Viral DNA was collected by filtering samples independently through a 0.22-μm-pore-size capsule filter (Millipore Sterivex-GV, PVDF [polyvinylidene difluoride] membrane) to remove most cellular organisms followed by collection of virus-sized particles on 25-mm-diameter 0.02-μm-pore-size Anotop aluminum oxide filters (Whatman). Sample volumes ranged from 375 to 960 ml. Filters were immediately frozen at –20°C in the field and then stored at –80°C at Université Laval until extraction.

Nutrient samples were analyzed as described previously by Laurion et al. (55) at l’Institut National de la Recherche Scientifique, Centre Eau-Terre-Environnement (Québec, Canada), while flow cytometry samples were processed at l’Université du Québec à Rimouski, Institut des sciences de la mer de Rimouski (Rimouski, Canada), as described previously by Brussaard et al. (56). Pigments were analyzed by high-performance liquid chromatography (HPLC) as described previously by Thaler et al. (57). Chlorophyll-*a* and its allomers were quantified by their absorbance at 450 nm and by fluorescence, zeaxanthin by absorbance at 450 nm, and bacteriochlorophyll-*e*-like pigments by absorbance at 467 nm. Total nucleic acids were extracted directly from individual Anotop filters using a Complete DNA & RNA purification kit (MasterPure; Epicentre) and the backflushing technique described previously by Mueller et al. (58). Libraries were prepared independently for all 15 samples with 10 ng of sheared DNA (350 to 400 bp) (Covaris M220 Ultrasonicator) using a NEBNext Ultra II library preparation kit (New England Biolabs). Paired-end sequencing (2 × 125 bp) was performed on an Illumina HiSeq 2500 system at the McGill University and Génome Quebec Innovation Centre (Montréal, Canada), yielding 125,403,906 reads (see Table S2 in the supplemental material).

10.1128/mSphere.00334-20.6 TABLE S2 Sequence processing details. Download Table S2, PDF file, 0.1 MB. Copyright © 2020 Labbé et al. 2020 Labbé et al. This content is distributed under the terms of the Creative Commons Attribution 4.0 International license.

### Sequence processing.

Initial read quality was assessed with FastQC (v.0.11.2 [59]). Adapter sequences were removed with Trimmommatic (v.0.36 [60]) in “readthrough” mode using a custom adapter file to supplement Illumina’s default adapter file. Reads were also trimmed to remove low-quality nucleotides (Phred score, <20) over the first and last 20 bases, and unpaired reads were discarded (Table S2). Error correction of base-calling was performed on paired reads with Karect (v.1.0) with default settings (61). The average length of the processed reads was 124 bp. Processed paired reads obtained independently from each sample were assembled *de novo* with MetaSPAdes (v.3.10.1) with default settings (62).

Viral sequences in the assembled data set were identified using VirSorter (v.1.0.3) and VirFinder (63, 64). VirSorter confidence categories 1 and 2 were kept for our final viral data set. Contigs that fell into category 3 or were rejected by VirSorter were subsequently analyzed with VirFinder, trained with a model that included eukaryotic viruses downloaded from the VirFinder GitHub site (https://github.com/jessieren/VirFinder). Contigs with a VirFinder confidence score greater or equal to 0.8 were added to the final viral data set. Only viral contigs longer than or equal to 2 kb were kept for further analysis (Table S2).

Viral contigs from the 15 independent samples were clustered together into vOTUs using ClusterGenomes software (v.1.1.3) on the iVirus.us platform (65). This program groups sequences that share an identity level of 95% over 85% of their length into a sequence cluster; the longest sequence in the cluster is then designated the vOTU. Processed quality reads from each sample were then individually mapped to the vOTUs with Bowtie2 (v.2.3.4.1 [66]) with the “end to end” parameter and SAMtools (v.1.8 [67]). These alignment files were then merged into a single distribution matrix showing the number of reads mapped to each vOTU in each sample. The program Read2Ref Mapper (v.1.1.0 [68]) was used to merge the files and normalize counts by vOTU sequence length (68), ultimately producing a vOTU table.

An unprocessed vOTU table was used for viral diversity and presence-absence analyses, while distance-based (beta diversity, neighbor-joining tree) and correlation analyses were performed on the basis of a vOTU table that was filtered to remove the least abundant 5% of vOTUs and those present in only one sample, resulting in the removal of 1,056 vOTUs. The table was then standardized using the total square root method (Hellinger) [decostand() in vegan{}] (69), and the data were log-transformed in R (R Development Core Team, v. 3.5.1).

### Data analysis.

All statistical analyses were performed in R, and *P* values are reported for statistically significant results only (α = 0.05). Limnological parameters and flow cytometry data were compared using the Kruskal-Wallis nonparametric test [kruskal.test() function in stats{}] to compare communities, as the assumptions for parametric tests were not met [tested with shapiro.test() and bartlett.test()]. Dunn’s test was used as a *post hoc* test using multiple-comparison correction (Bonferroni correction) in the FSA{} package (70).

Community-level diversities were compared across depths as well as across lake strata (mixolimnion, metalimnion, and monimolimnion). Metadata collinearity was verified in vegan{} by the use of backward selection and the vif.cca() function. Adjusted *R*^2^ values were calculated in vegan{} with the RsquareAdj() function. Sample-wise community comparisons (beta diversity) were performed using the Bray-Curtis dissimilarity index with phyloseq{}. Ordinations were visualized with principal-coordinate analysis (PCoA) and constrained analysis of principal coordinates (CAP). Differences between centroids of groups were assessed using permutational multivariate analysis of variance (PERMANOVA) with adonis(), and within-group homogeneity of dispersions was verified with betadisper(), both in vegan{}. A sample replicate neighbor-joining tree was created using the Bray-Curtis dissimilarity index with the ape{} package (71) in R. Overlap in vOTUs across lake layers is shown in a Venn diagram computed using Venny 2.0 (72) and was plotted using the VennDiagram{} package in R (73). A correlation matrix of metadata variables was produced with cor() in stats{} and visualized with corrplot{} (74). Boxplot graphs (showing medians and whiskers of the 25% and 75% quartiles) were produced with ggplot2{} (75) on the basis of the number of vOTUs at each depth. As normality and heteroscedasticity were not respected, we used the nonparametric Kruskal-Wallis rank sum test to compare diversities between depths as well as layers (mixolimnion, metalimnion, and monimolimnion) and used Dunn’s test as a *post hoc* test to correct for multiple comparisons.

The WGCNA{} R package (76) was used to determine correlations between vOTUs and environmental parameters, as described previously by Guidi et al. (77). The vOTUs were first clustered into modules based on their cooccurrence within samples using the Pearson correlation. Next, a second Pearson correlation, with *P* values corrected for multiple comparisons (Bonferroni) by p.adjust(), was used to determine how environmental parameters correlated with each module’s occurrence (see Fig. S2 in the supplemental material). Correlations with *P* values of ≤0.001 were considered highly significant, and vOTUs belonging to the corresponding modules were used for further analysis. Step-wise positive selection was used to identify covarying variables, which were then removed from the final WGCNA.

The vOTUs from modules significantly correlated with a lake stratum were further investigated using BLASTx (78). The complete IMG/VR protein database (32) was used to explore the similarity between the vOTUs from this study and viral sequences from metagenomes collected throughout the globe. BLASTx results were filtered for top hits with an E value lower than 0.01, an alignment of a minimum of 65 amino acids, and ≥85% identity, and the top hit (*max_target_seqs* option set to 1) was used. The IMG/VR “taxonIDs” metadata were then used to group these results into manually curated habitat types. This threshold was selected on the basis of an iterative process in which we progressively lowered the percent identity value until a threshold (85%) was reached that resulted in at least one match for each WGCNA module. vOTUs that were classified as circular by VirSorter were filtered to select sequences that were >30 kb. Of these, 10 vOTUs were selected based on the following criteria: they were classified in VirSorter category 1 or 2, contained hallmark viral genes, and were part of a WGCNA module that was significantly correlated with an important biological or limnological variable (see Table S3 for a list of characteristics of the VAGs selected). These VAGs were annotated using Prokka (v1.13.7 [79]) with the viral kingdom option. Gene annotations based on Swiss-Prot reference clusters (80) for each VAG can be found in Table S3. Genome maps were made for 5 VAGs with SnapGene software. Classification of the VAGs was determined with ViPTree (v1.9 [31]).

10.1128/mSphere.00334-20.7 TABLE S3 UniProtKB-Prokka annotation and metadata for 10 circular viral assembled genomes (VAGs). Download Table S3, PDF file, 0.9 MB. Copyright © 2020 Labbé et al. 2020 Labbé et al. This content is distributed under the terms of the Creative Commons Attribution 4.0 International license.

We used Libra (34) to examine the similarities between viral reads of samples collected from the depths of 5, 15, and 40 m of Lake A (replicate III only), those of samples from the Arctic Ocean (33) collected approximately 100 km from Utkiagvik, AK. (station 196, 5 m; ENA accession number ERS1309397), and those of samples from the boundary between the Chukchi Sea and the Arctic Basin (station 194) (5 and 35 m; ENA accession numbers ERS1309308 and ERS1309369, respectively) and of samples from Ace Lake (29), a meromictic lake in the Vestfold Hills, Antarctica (depths of 5, 12.7, and 23 m; MG-Rast accession numbers mgm4443684.3, mgm4443681.3, and mgm4443683.3). For both meromictic lakes, the depths selected were considered representative of the three strata of each lake. Viral reads were identified in the following manner: for each sample, reads were assembled with Megahit (81) (default settings), viral contigs were identified with VirFinder (EPV model; all contigs with a score of ≥0.9 were retained), and raw reads were then mapped to viral contigs using Bowtie2 (66) with the option –al-conc. Viral reads were then compared using Libra (34) with *k*-mers of 20 bp, using the default cosine similarity scoring and logarithmic weighting parameters. The distance matrix produced by Libra with the as.dendrogram() function in the standard R stats{} package was used to create Fig. 7.

### Data availability.

High-quality processed reads are available in the Sequence Read Archive of the National Center for Biotechnology Information (SRA-NCBI) under BioProject accession number PRJNA545459 or individually as listed in Table S2. The vOTU abundance table and the vOTU sequence files are now available on the ViDEL GitHub site (https://github.com/LabViDEL/Viral-diversity-in-Arctic-meromictic-lake). Data from Ace Lake (28) and the Tara Polar Expedition (33) were downloaded from MG-RAST (www.mg-rast.org [82]) and the European Nucleotide Archive (PRJEB9742), respectively, on 4 April 2019. Environmental metadata for Lake A are archived in Nordicana D (83).
