# Supplementary figures and images for: Extreme Viral Partitioning in a Marine-Derived High Arctic Lake
Source: mSphere. 2020 May 13;5(3):e00334-20. doi: 10.1128/mSphere.00334-20 (PMC7227771; doi:10.1128/mSphere.00334-20)

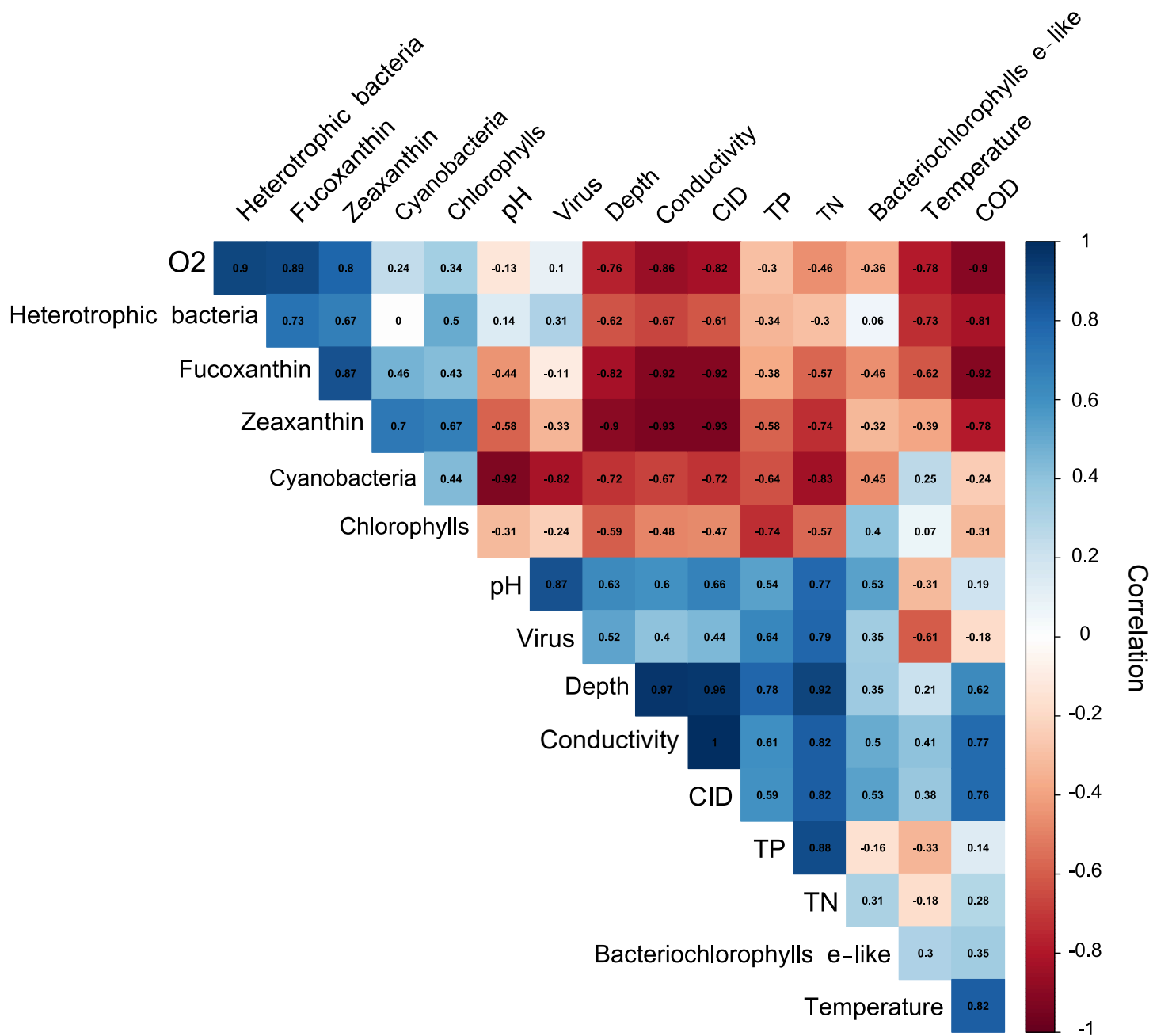

FIGURE S1

Supplement: FIG S1 [file mSphere.00334-20-sf001.pdf]

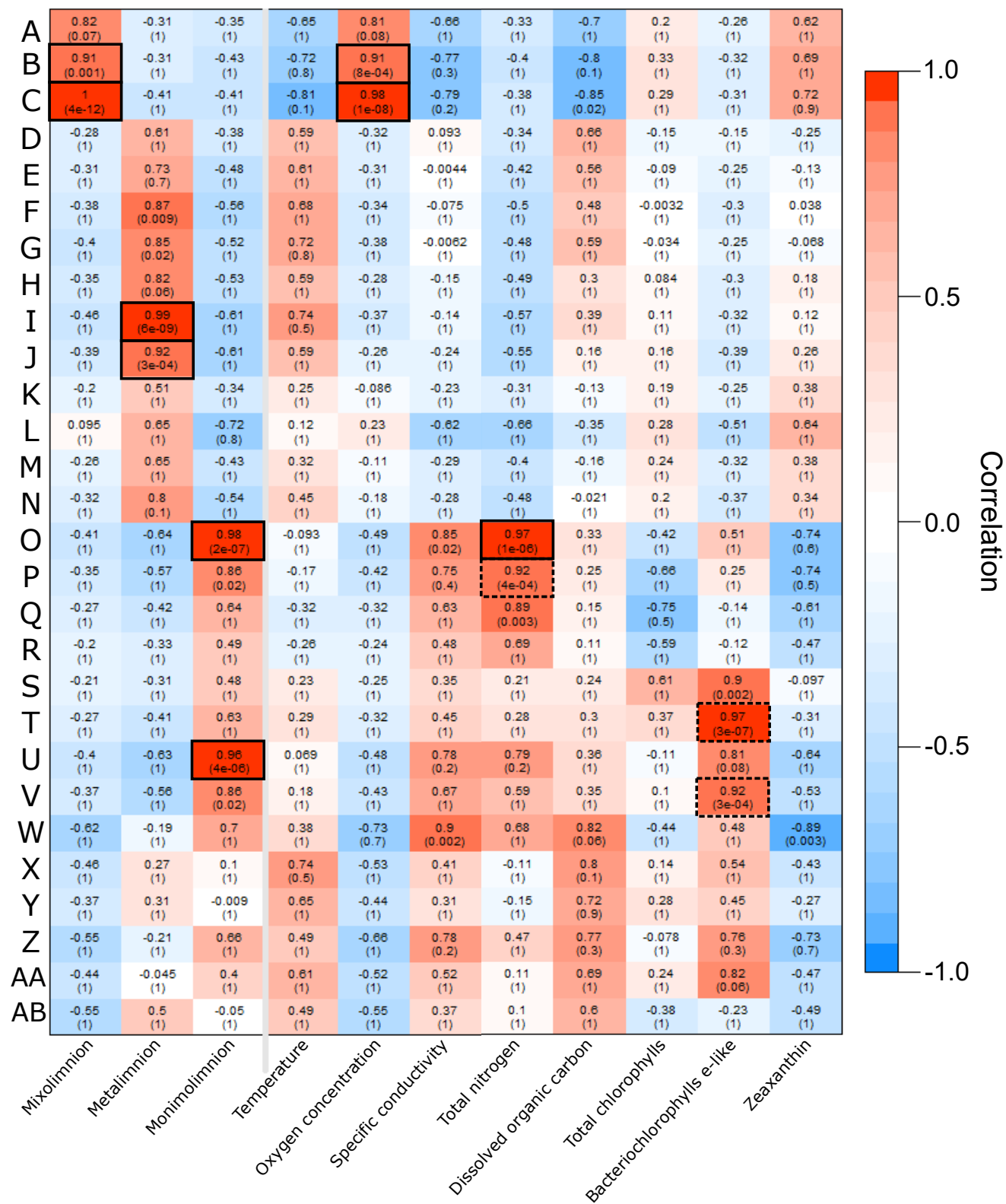

FIGURE S2

Supplement: FIG S2 [file mSphere.00334-20-sf002.pdf]

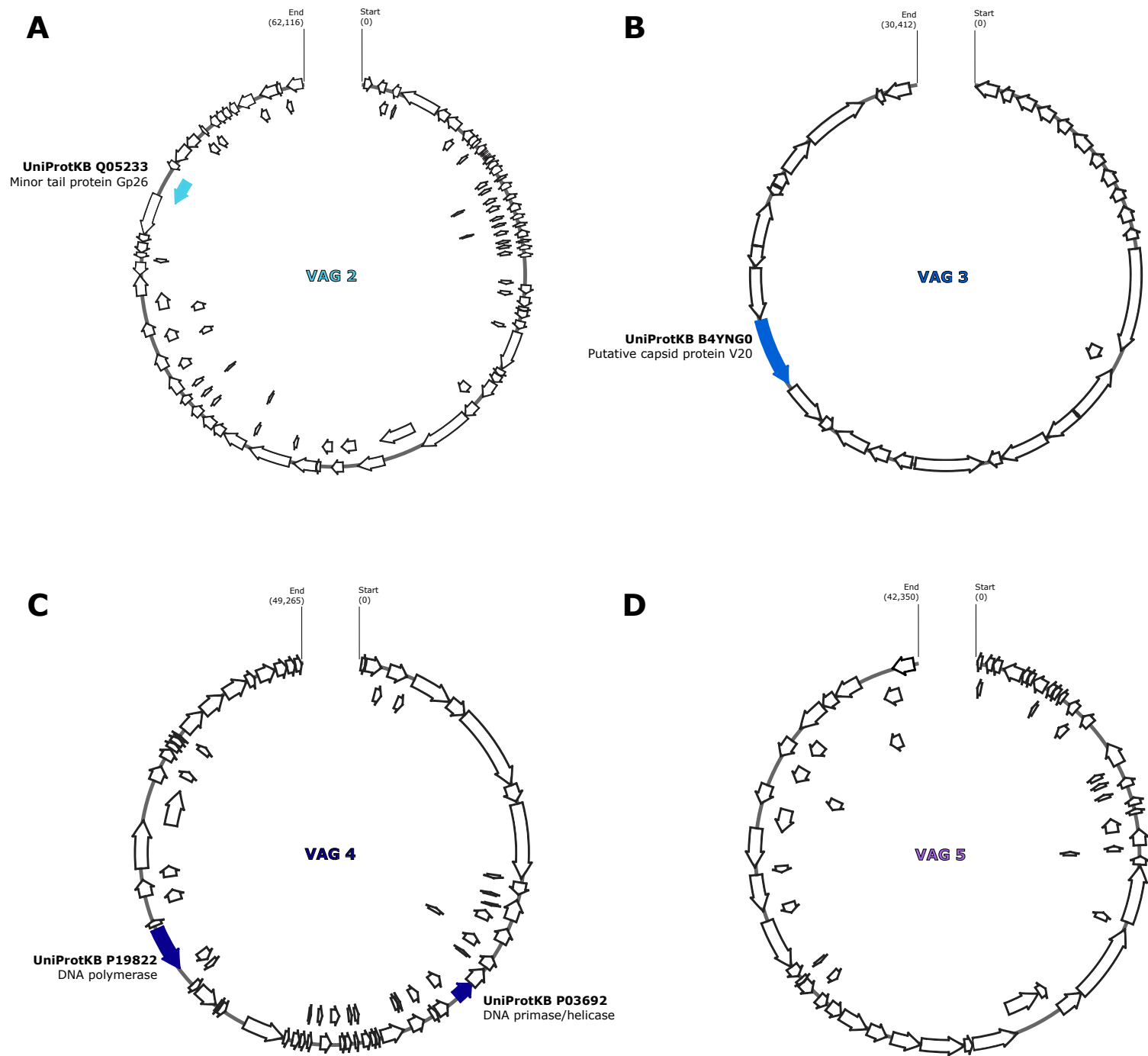

**FIGURE S3**

Supplement: FIG S3 [file mSphere.00334-20-sf003.pdf]
