# Supplementary material for: Extreme Viral Partitioning in a Marine-Derived High Arctic Lake
Source: mSphere. 2020 May 13;5(3):e00334-20. doi: 10.1128/mSphere.00334-20 (PMC7227771; doi:10.1128/mSphere.00334-20)
Supplement: TABLE S1 [file mSphere.00334-20-st001.pdf]

TABLE S1. Modules with significant correlations to environmental variables based on WGCNA.

Module	Size (vOTUs)	Water-column layer	Environmental variable
B	220	Mixolimnion	Oxygen
C	287	Mixolimnion	Oxygen
I	49	Metalimnion	N/A
J	290	Metalimnion	N/A
O	1538	Monimolimnion	Total nitrogen
P	61	N/A	Total nitrogen
T	1176	N/A	High bacteriochlorophylls *e*-like concentration
U	710	Monimolimnion	N/A
V	130	N/A	High bacteriochlorophylls *e*-like concentration

N/A: no significant correlation was observed
