# Supplementary material for: Extreme Viral Partitioning in a Marine-Derived High Arctic Lake
Source: mSphere. 2020 May 13;5(3):e00334-20. doi: 10.1128/mSphere.00334-20 (PMC7227771; doi:10.1128/mSphere.00334-20)
Supplement: FIG S4 [file mSphere.00334-20-sf004.pdf]

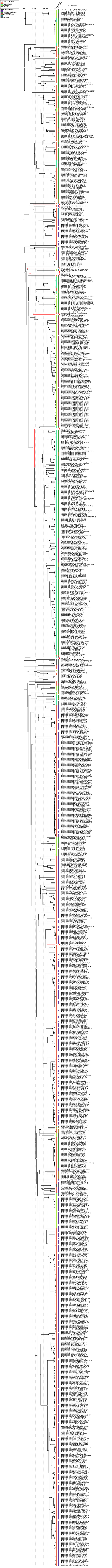
Left line: Virus family
Siphoviridae (822)
Podoviridae (223)
Myoviridae (191)
Others (7)
Right line: Host group
Actinobacteria (553)
Gammaproteobacteria (328)
Cyanobacteria (111)
Alphaproteobacteria (44)
Firmicutes (11)
Others (82)
0.001
0.005
0.01
0.05
0.1
0.5
Virus family
Host group
1277 sequences
