# Supplementary material for: Extreme Viral Partitioning in a Marine-Derived High Arctic Lake
Source: mSphere. 2020 May 13;5(3):e00334-20. doi: 10.1128/mSphere.00334-20 (PMC7227771; doi:10.1128/mSphere.00334-20)
Supplement: TABLE S3 [file mSphere.00334-20-st003.pdf]

**TABLE S3**. UniProtKB-Prokka annotation and metadata for 10 circular viral assembled genomes (VAGs).
NOTE: Data for only non-hypothetical proteins presented here; bold indicates VAGs that were presented in Figure 5 of the main text

| VAG# | vOTU ID | WGCNA module | Water-column distribution | Genome size (bp) | Total ORFs | Contig hit | EC number | UniProtKB code | UniProtKB gene ID | UniProtKB viral ID | UniProtKB viral lineage |
|---|---|---|---|---|---|---|---|---|---|---|---|
| 6 | VIRSorter_NODE_47_length_54735_cov_16_1727 | *none* | abundant at 5m, present at 15m | 54,735 | 91 | KNFAEGHP_00023 | NA | Q05235 | Minor tail protein Gp28 | *Mycobacterium phage L5 (Mycobacteriophage L5)* | Viruses › Caudovirales › Siphoviridae › Fromanvirus |
| | | | | | | KNFAEGHP_00051 | 2.7.7.- | P21442 | Integrase | *Haemophilus phage HP1 (strain HP1c1) (Bacteriophage HP1)* | Viruses › Caudovirales › Myoviridae › Peduovirinae › Hpunavirus › Haemophilus phage HP1 |
| | | | | | | KNFAEGHP_00082 | NA | Q9ZX29 | Probable transcriptional regulator WhiBTM4 | *Mycobacterium phage TM4 (Mycobacteriophage TM4)* | Viruses › Caudovirales › Siphoviridae › Timquatrovirus › Mycobacterium virus TM4 |
| 7 | VIRSorter_NODE_11_length_101329_cov_28_0366 | *none* | only present at 5m, largest genome (>100kb), 150-160 ORFs | 101,329 | 159 | GCLMJFHJ_00029 | NA | P25480 | Probable portal protein | *Escherichia phage P2 (Bacteriophage P2)* | Viruses › Caudovirales › Myoviridae › Peduovirinae › Peduovirus |
| | | | | | | GCLMJFHJ_00058 | 1.17.4.1 | O64174 | Ribonucleoside-diphosphate reductase subunit beta | *Bacillus phage SPbeta (Bacillus phage SPBc2) (Bacteriophage SP-beta)* | Viruses › Caudovirales › Siphoviridae › Spbetavirus |
| | | | | | | GCLMJFHJ_00059 | 1.17.4.1 | Q76RD8 | Ribonucleoside-diphosphate reductase large subunit | *Vaccinia virus (strain Ankara) (VACV)* | Viruses › Poxviridae › Chordopoxvirinae › Orthopoxvirus › Vaccinia virus |
| **5** | **VIRSorter_NODE_30_length_42405_cov_14_8062** | **J (metalimnion)** | **present everywhere but 5m, very abundant at 15m** | **42,350** | **63** | ***none*** | | | | | |
| 8 | VIRSorter_NODE_4_length_87432_cov_13_5877 | N | present only in metalimnion, especially at 15m | 87,432 | 155 | LOFIEDBH_00018 | 3.4.-.- | P85500 | Capsid polyprotein | *Pseudomonas phage PAJU2* | Viruses › Caudovirales › Siphoviridae › Detrevirus › unclassified Detrevirus |
| | | | | | | LOFIEDBH_00019 | 3.4.21.- | Q6QGD7 | Prohead protease | *Escherichia phage T5 (Enterobacteria phage T5)* | Viruses › Caudovirales › Siphoviridae › Tequintavirus |
| 9 | VIRSorter_NODE_13_length_64037_cov_26_883 | T (bacterio-chlorophyll-e-likes pigments) | unique to 28m, pretty abundant | 64,037 | 91 | KBGDBGMK_00072 | NA | P68928 | Terminase small subunit | *Bacillus phage SF6 (Bacteriophage SF6)* | Viruses › Caudovirales › Siphoviridae › unclassified Siphoviridae |
| | | | | | | KBGDBGMK_00078 | NA | P06153 | Immunity repressor protein | *Bacillus phage phi105 (Bacteriophage phi-105)* | Viruses › Caudovirales › Siphoviridae › unclassified Siphoviridae |
| | | | | | | KBGDBGMK_00088 | 2.7.7.7 | P19822 | DNA polymerase | *Escherichia phage T5 (Enterobacteria phage T5)* | Viruses › Caudovirales › Siphoviridae › Tequintavirus |
| | | | | | | KBGDBGMK_00089 | NA | P04526 | Sliding-clamp-loader gp44 subunit | *Enterobacteria phage T4 (Bacteriophage T4)* | Viruses › Caudovirales › Myoviridae › Tevenvirinae › Tequatrovirus |
| **2** | **VIRSorter_NODE_8_length_62171_cov_111_284** | ***none*** | **unique to metalimnion, especially at 15m** | **62,116** | **106** | **CGDLDOEA_00094** | **NA** | **Q05233** | **Minor tail protein Gp26** | ***Mycobacterium phage L5 (Mycobacteriophage L5)*** | **Viruses › Caudovirales › Siphoviridae › Fromanvirus** |
| 10 | VIRSorter_NODE_10_length_54657_cov_20_8832 | *none* | unique to metalimnion | 54,657 | 72 | NKNDGJMO_00050 | 3.2.1.17 | P11187 | Endolysin | *Bacillus phage phi29 (Bacteriophage phi-29)* | Viruses › Caudovirales › Podoviridae › Picovirinae › Salasvirus |
| | | | | | | NKNDGJMO_00065 | 2.7.7.7 | P19822 | DNA polymerase | *Escherichia phage T5 (Enterobacteria phage T5)* | Viruses › Caudovirales › Siphoviridae › Tequintavirus |
| | | | | | | NKNDGJMO_00069 | 3.1.11.1 | P04536 | Exodeoxyribonuclease | *Enterobacteria phage T4 (Bacteriophage T4)* | Viruses › Caudovirales › Myoviridae › Tevenvirinae › Tequatrovirus |
| | | | | | | NKNDGJMO_00071 | 3.1.22.- | P03015 | Serine recombinase gin | *Escherichia phage Mu (Bacteriophage Mu)* | Viruses › Caudovirales › Myoviridae › Muvirus |
| **4** | **VIRSorter_NODE_29_length_49320_cov_59_3851** | **U (monimo-limnion)** | **abundant at 28 and 40m** | **49,265** | **76** | **ICIEGKNE_00023** | **2.7.7.-** | **P03692** | **DNA primase/helicase** | ***Enterobacteria phage T7 (Bacteriophage T7)*** | **Viruses › Caudovirales › Podoviridae › Autographivirinae › Teseptimavirus › Escherichia virus T7** |
| | | | | | | **ICIEGKNE_00055** | **2.7.7.7** | **P19822** | **DNA polymerase** | ***Escherichia phage T5 (Enterobacteria phage T5)*** | **Viruses › Caudovirales › Siphoviridae › Tequintavirus** |
| **1** | **VIRSorter_NODE_36_length_44859_cov_34_6262** | **T (bacterio-chlorophyll-e-likes pigments)** | **only present at 28m** | **44,805** | **50** | **DHLCIPJL_00019** | **3.6.4.-** | **P11107** | **Probable helicase D10** | ***Escherichia phage T5 (Enterobacteria phage T5)*** | **Viruses › Caudovirales › Siphoviridae › Tequintavirus** |
| | | | | | | **DHLCIPJL_00021** | **2.7.7.6** | **P00573** | **T7 RNA polymerase** | ***Enterobacteria phage T7 (Bacteriophage T7)*** | **Viruses › Caudovirales › Podoviridae › Autographivirinae › Teseptimavirus › Escherichia virus T7** |
| | | | | | | **DHLCIPJL_00025** | **NA** | **P03728** | **Portal protein** | ***Enterobacteria phage T7 (Bacteriophage T7)*** | **Viruses › Caudovirales › Podoviridae › Autographivirinae › Teseptimavirus › Escherichia virus T7** |
| | | | | | | **DHLCIPJL_00028** | **NA** | **P19727** | **Minor capsid protein** | ***Enterobacteria phage T7 (Bacteriophage T7)*** | **Viruses › Caudovirales › Podoviridae › Autographivirinae › Teseptimavirus › Escherichia virus T7** |
| | | | | | | **DHLCIPJL_00035** | **NA** | **P03746** | **Tail tubular protein gp11** | ***Enterobacteria phage T7 (Bacteriophage T7)*** | **Viruses › Caudovirales › Podoviridae › Autographivirinae › Teseptimavirus › Escherichia virus T7** |
| | | | | | | **DHLCIPJL_00036** | **NA** | **P03747** | **Tail tubular protein gp12** | ***Enterobacteria phage T7 (Bacteriophage T7)*** | **Viruses › Caudovirales › Podoviridae › Autographivirinae › Teseptimavirus › Escherichia virus T7** |
| | | | | | | **DHLCIPJL_00041** | **3.1.-.-** | **P03694** | **Terminase, large subunit gp19** | ***Enterobacteria phage T7 (Bacteriophage T7)*** | **Viruses › Caudovirales › Podoviridae › Autographivirinae › Teseptimavirus › Escherichia virus T7** |
| **3** | **VIRSorter_NODE_144_length_30467_cov_60_9971** | **B (mixolimnion & oxygen)** | **very abundant at 5m, present at 15m** | **30,412** | **33** | **FIEOIHON_00024** | **NA** | **B4YNG0** | **Putative capsid protein V20** | ***Sputnik virophage*** | **Viruses › Lavidaviridae › Sputnikvirus › Mimivirus-dependent virus Sputnik** |
