# Supplementary material for: Extreme Viral Partitioning in a Marine-Derived High Arctic Lake
Source: mSphere. 2020 May 13;5(3):e00334-20. doi: 10.1128/mSphere.00334-20 (PMC7227771; doi:10.1128/mSphere.00334-20)
Supplement: TABLE S2 [file mSphere.00334-20-st002.pdf]

TABLE S2. Sequence processing details.

Sample	Sequencing	Quality check [a]		Assembly		Viral contig identification		Size filtering [b]	vOTU mapping
	Read count	Read count	Accession no.	Contig count	N50 (bp)	Contig count	N50 (bp)	Contig count	Percentage of recruited reads [c]
5m triplicate	19,384,960	19,319,104		566,849	761	8121	11,147	6745	57.70
AI05	2,211,670	2,182,401	SAMN11892756	72,169	395	569	8984	430	60.81
AII05	9,298,996	9,295,973	SAMN11892757	249,889	1045	4055	12,785	3402	58.28
AIII05	7,874,294	7,840,730	SAMN11892758	244,791	843	3497	11,672	2913	54.02
15m triplicate	19,894,901	19,768,736		991,655	442.7	4421	10,674	3911	44.80
AI15	1,822,241	1,732,047	SAMN11892759	90,978	297	365	10,675	312	56.79
AII15	11,520,785	11,513,604	SAMN11892760	520,896	551	2381	10,821	2154	39.64
AIII15	6,551,875	6,523,085	SAMN11892761	379,781	480	1675	10,526	1445	37.97
20m triplicate	32,434,003	32,178,670		1,387,312	393.3	6728	11,355.3	6179	55.88
AI20	13,076,691	13,050,214	SAMN11892762	534,157	412	2747	11,622	2516	55.36
AII20	10,267,801	10,071,064	SAMN11892763	441,955	377	1872	10,986	1729	54.96
AIII20	9,089,511	9,057,392	SAMN11892764	411,200	391	2109	11,458	1934	57.32
28m triplicate	27,862,532	27,365,617		1,387,385	387	5860	12,830.7	5458	44.77
AI28	9,809,644	9,642,476	SAMN11892765	471,816	378	1938	12,903	1796	46.62
AII28	9,624,201	9,534,513	SAMN11892766	440,435	407	2122	13,091	1984	46.20
AIII28	8,428,687	8,188,628	SAMN11892767	475,134	376	1800	12,498	1678	41.49
40m triplicate	26,327,065	26,075,334		2,475,415	379.7	8135	9167.7	7311	22.40
AI40	7,759,734	7,705,466	SAMN11892768	753,317	395	2586	8889	2323	24.10
AII40	10,783,327	10,685,294	SAMN11892769	955,348	389	3622	8895	3259	22.50
AIII40	7,784,004	7,684,574	SAMN11892770	766,750	355	1927	9719	1729	20.59
All [d]	125,903,461	124,707,461	PRJNA545459	6,808,616	472.7	33,265	11,034.9	29,604	45.11

[a] Quality check involves trimming for adapters and low-quality bases, removal of unpaired reads, and error-correction.

[b] Filtered to remove contigs smaller than 2000bp

[c] Percentage of quality filtered reads from each sample that have been recruited by the vOTU assemblage (which merges viral contigs from all samples into a single assemblage)

[d] Counts are given as sums, N50 and percentage are given as mean. Accession number is for SRA BioProject.
